# Easy conic intersection with the common self-polar triangle

**DOI:** 10.1371/journal.pone.0340348

**Published:** 2026-07-17

**Authors:** Michela Mancini, John A. Christian

**Affiliations:** Guggenheim School of Aerospace Engineering, Georgia Institute Of Technology, Atlanta, Georgia, United States of America; Newcastle University, UNITED KINGDOM OF GREAT BRITAIN AND NORTHERN IRELAND

## Abstract

Intersecting two conics is a classical problem that is frequently encountered in many different areas of science, engineering, and art. For example, under perspective projection (e.g., in camera images), any degree-two curve (a conic) or surface (a quadric) projects to a conic. This is important since polynomials of degree two are commonly used to approximate the contour or surface of many real-world objects. This manuscript describes a simple solution to the conic intersection problem using a change of projective coordinates. Exploiting the properties of self-polar triangles, we show how to reduce the task to simply solving an eigenvalue problem of degree three and a quadratic equation in one variable. The self-polar triangle method provides an attractive alternative to more common methods, especially in settings that require explicit algorithmic solutions.

## Introduction

Conics are a ubiquitous shape in nature. In space, conics describe the shape of Keplerian orbits [1,2] and the rims of impact craters on celestial bodies [3]. On Earth, conics describe the shape of famous architectural works (e.g., the roof of the Tycho Brahe Planetarium in Copenhagen) and everyday objects (e.g., wheels, rim of a coffee mug, bottle caps). In biology, conics describe the cross-section of a blood vessel or the shape of the iris and pupil of the human eye. Although these (and other) real-world objects are not always perfect conics, this approximation is often quite good. Given the pervasive nature of conics in the world, it is common to desire knowledge of the intersection points of two such curves. For example, conic intersections are used to identify crater patterns on the lunar surface [4], detect the orientation of a face [5], or estimate camera attitude from 2D-to-3D point correspondences [6]. Regardless of the application, the study of this classical problem presents a number of delightful geometric results.

The intersection of two conics is simply the solution to a pair of degree two polynomials in two variables. Computing the solution to this problem is trivial in environments where modern computer algebra packages are available (e.g., Maple, Mathematica). Unfortunately, however, high-performance or safety-critical embedded systems—such as those encountered in many robotics or aerospace applications—are usually not permitted to rely on computer algebra softwares. Instead, explicit solutions with performance guarantees are required. When such explicit solutions are required, the most popular approach for intersecting two conics proceeds by (1) finding the degenerate conic consisting of two lines passing through the points of intersection and then (2) intersecting these lines with one of the conics [7,8]. There are also other approaches that make use of other methods, such as Gröbner bases [9], geometric algebra [10], or that reformulate the problem in terms of a degree-four equation in a single variable, which can then be solved analytically [11]. At some level of abstraction, all of these approaches solve the same fundamental problem and it is a small task to reinterpret any one method in terms of any of the others. Despite being equivalent, however, there are definite algorithmic advantages to the practical implementation of some interpretations.

In this work we consider a particular interpretation of the conic intersection problem that permits an especially simple algorithm that can be directly written into software. This interpretation is based on a convenient projective transformation built from the two conics’ common self-polar triangle.

The self-polar triangles of a conic are a well-studied topic with thorough discussions in modern textbooks [12,13]. The *common* self-polar triangle between two conics has recently been explored for camera calibration [14–16] and for computing the homography between two images of the same ellipse [17]. The algorithm presented in this work is built around a different use of the common self-polar triangle that has not yet appeared in the contemporary literature.

The notion that self-polar triangles may produce a transformation helpful for computing conic intersections is not new. Indeed, that the common self-polar triangle could be used to find conic intersections was suggested as early as the mid 1800s [18,19]. Nevertheless, these notions were never extended into practical algorithms and are not well-known outside of a few specialized corners of mathematics. To the best of the authors’ knowledge, more recent discussions of conic intersections by self-polar triangles than in Refs. [18,19] (which are in German, not English) are not amongst the corpus of commonly-available literature. This short article is intended to rescue this interpretation from (relative) obscurity and to show how it can be specialized into a practical and easy-to-implement algorithm for computing conic intersections. The algorithm presented here has been implemented in MATLAB and is available through the open-source SONIC toolkit [20] (The SONIC toolkit may be found at https://github.com/opnavlab/sonic).

### Mathematic preliminaries

Consider a plane containing a conic in general position. Its equation can be written as a polynomial of degree two:


$$\begin{array}{c} \;Ax^{2}+Bxy+Cy^{2}+Dx+Ey+F=0 \end{array}$$ (1)

Using projective coordinates, the same equation can be recast in matrix form. In fact, if we let $x\propto [x\;y\;1{]}^{T}\in {\mathbb{P}}^{2}$ be the coordinates of a point in the projective plane, the previous equation can be expressed as


$$\begin{array}{c} x^{T}[\begin{array}{ccc} A & B/2 & D/2\\ B/2 & C & E/2\\ D/2 & E/2 & F \end{array}]x=x^{T}\;C\;x=0 \end{array}$$ (2)

so that we can associate the conic to the conic matrix C of ambiguous scale


$$\begin{array}{c} C\propto [\begin{array}{ccc} A & B/2 & D/2\\ B/2 & C & E/2\\ D/2 & E/2 & F \end{array}] \end{array}$$ (3)

We observe that the conic matrix C is guaranteed to always be full-rank for any non-singular conic—including circles, ellipses, parabolas, and hyperbolas. The proportionality relationship captures the scale ambiguity of C since any non-zero scaling describes the same conic.

The matrix C also defines a *polarity relationship* with respect to the conic. Recalling that both points and lines can be expressed as 3-tuples in the projective plane using homogeneous coordinates (see [12] for more details on the duality in ${\mathbb{P}}^{2}$), any point x on the plane can be associated to its *polar line* u with respect to that conic according to


$$\begin{array}{c} u\propto Cx \end{array}$$ (4)

Similarly, if we let $C^{*}\propto C^{-1}$, any line u can be associated to its polar point x using


$$\begin{array}{c} x\propto C^{*}u \end{array}$$ (5)

### Change of projective coordinates

Consider any four points $p_{i}$ (*i* = 0, 1, 2, 3) in the projective plane, no three of which are collinear. Such a set of points can be used to define a new coordinate system in ${\mathbb{P}}^{2}$. In fact, we can find a non-singular linear transformation that maps the reference points $e_{0}^{T}\propto [1\;0\;0]$, $e_{1}^{T}\propto [0\;1\;0]$, $e_{2}^{T}\propto [0\;0\;1]$ and the unit point $e_{3}^{T}\propto [1\;1\;1]$ to the four points $p_{0}$, $p_{1}$, $p_{2}$ and $p_{3}$, respectively. This mapping is represented by the homography matrix H such that [12]


$$\begin{array}{c} He_{i}=\lambda_{i}p_{i}\;i=0,1,2 \end{array}$$ (6)

where $\lambda_{i}$ are unknown scale factors that need to be determined. The further condition


$$\begin{array}{c} He_{3}=\lambda_{3}p_{3} \end{array}$$ (7)

fixes the scaling once the parameter $\lambda_{3}$ has been specified. Taking advantage of our simple choices for $e_{0},e_{1},e_{2}$, the first of these two equations implies that the homography matrix H is


$$\begin{array}{c} H\propto \left[\lambda_{0}p_{0}\;\lambda_{1}p_{1}\;\lambda_{2}p_{2}\right] \end{array}$$ (8)

Selecting a scaling of $\lambda_{3}=1$, we can re-write Eq. (7) simply as [12]


$$\begin{array}{c} {}[\begin{array}{ccc} \lambda_{0}p_{0} & \lambda_{1}p_{1} & \lambda_{2}p_{2} \end{array}][\begin{array}{c} 1\\ 1\\ 1 \end{array}]=p_{3} \end{array}$$ (9)

which is the same as


$$\begin{array}{c} {}[\begin{array}{ccc} p_{0} & p_{1} & p_{2} \end{array}][\begin{array}{c} \lambda_{0}\\ \lambda_{1}\\ \lambda_{2} \end{array}]=p_{3} \end{array}$$ (10)

Since $p_{0}$, $p_{1}$ and $p_{2}$ are not collinear, the matrix on the left-hand side is always invertible. Thus, we can solve directly for the $\lambda_{0},\lambda_{1},\lambda_{2}$, which allows for the computation of H from Eq. (8). Given a point $x^{\prime }$ expressed in the projective coordinate system defined by $\left\{p_{i}\right\}$, we can recover the corresponding coordinates x associated with the basis $\left\{e_{i}\right\}$ by applying


$$\begin{array}{c} x\propto Hx^{\prime } \end{array}$$ (11)

## Intersecting two conics using a self-polar triangle

In this section, we will show how to perform conic intersection using the concept of self-polar triangles. The main approach presented here is valid for any configuration where the two conics have at least one common self-polar triangle. This is almost always true in practice. The one exception is when the conics are tangent at a single point, but this special case is easily recognized and solved. The pseudocode for the corresponding algorithm is provided in Algorithm 1.

### Two conics in general position

Given three points in the plane, they are said to form the vertices of a self-polar triangle with respect to a conic if the polar line of each vertex is the line passing through the remaining two vertices. Any four distinct points on a conic may be used to form a quadrangle. By constraining these points to be on a conic, it may be shown that the quadrangle’s diagonal triangle will be a self-polar triangle [12].

While there are an infinite number of self-polar triangles for a given conic (since there are an infinite number of quadrangles from 4-tuples of distinct points on the conic), two distinct conics intersecting in four distinct points (over the complex numbers) can have only one self-polar triangle in common. In other words, there are only three points in the projective plane that form a triangle that is self-polar for both the conics [12,13]. If we let these vertices be the reference points of a new projective coordinate system, the two conic equations are greatly simplified, and their intersection can be performed easily.

**Fig 1:**
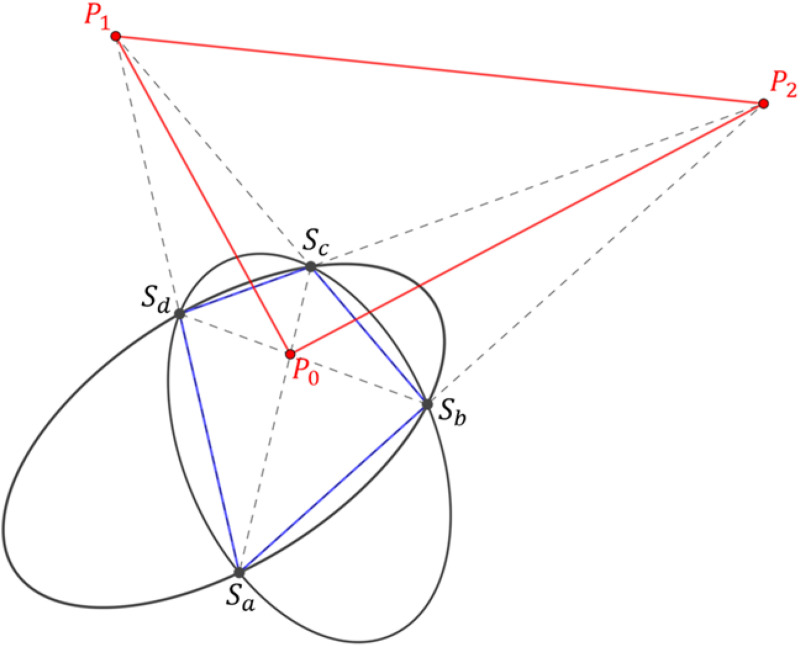
The points of intersection between non-consecutive edges of the quadrilateral $S_{a}S_{b}S_{c}S_{d}$ are the vertices of the unique triangle that is self-polar for both the two conics.

To visualize the common self-polar triangle, let $S_{a}$, $S_{b}$, $S_{c}$ and $S_{d}$ be the points of intersection of the two conics $C_{1}$ and $C_{2}$. The vertices of the unique common self-polar triangle can be found by intersecting the lines joining non-consecutive sides of the quadrilateral $S_{a}S_{b}S_{c}S_{d}$ [12]. This is illustrated in Fig 1 for the case of four real intersection points.

When the vertices of a conic’s self-polar triangle are used as reference points of the projective coordinate system, the conic matrix is transformed to a diagonal matrix. This can be easily seen if we consider that the reference points in the new frame have coordinates


$$\begin{array}{c} p_{0}^{\prime }\propto [\begin{array}{c} 1\\ 0\\ 0 \end{array}]\;p_{1}^{\prime }\propto [\begin{array}{c} 0\\ 1\\ 0 \end{array}]\;p_{2}^{\prime }\propto [\begin{array}{c} 0\\ 0\\ 1 \end{array}] \end{array}$$ (12)

Since the line passing through $p_{l}^{\prime }$ and $p_{m}^{\prime }$ has coordinates $\ell_{i}\propto p_{l}^{\prime }\times p_{m}^{\prime }$ and $p_{l}^{\prime }\times p_{m}^{\prime }\propto p_{i}^{\prime }$, the polarity relationship imposes that


$$\begin{array}{c} p_{i}^{\prime }\propto C^{\prime }p_{i}^{\prime } \end{array}$$ (13)

for all i∈{0,1,2}, that can only happen when $C^{\prime }$ is diagonal. If a triangle is self-polar for two conics, the change of coordinates will simultaneously diagonalize the conic matrices.

In order to construct the matrix H, we need to determine the vertices of the self-polar triangle $p_{0}$, $p_{1}$ and $p_{2}$ in the original coordinate system. Given the definition of self-polar triangle, we know that the polar line of any vertex passes through the other two. Since the triangle is self-polar for the two conics, this polarity relationship must hold for both, so that we have


$$\begin{array}{c} C_{1}p_{i}\propto C_{2}p_{i}\;i=0,1,2 \end{array}$$ (14)

Introduce the unknown scaling factor $\mu_{i}$ to remove the proportionality, yielding the eigenvalue problem


$$\begin{array}{c} \left(C_{2}^{*}C_{1}-\mu_{i}{\text{I}}_{3\times 3}\right)p_{i}=\text{0} \end{array}$$ (15)

We can conclude that the vertices of the common self-polar triangle are the eigenvectors of the matrix $C_{2}^{*}C_{1}$. Once the coordinates of the reference points have been determined, we can freely set the unit point $p_{3}^{T}=[1\;1\;1]$ so long as no three of these points are collinear.

These values of $p_{0},p_{1},p_{2},p_{3}$ may be used to construct the homography H using the procedure from the Section *Change of projective coordinates*. After the transformation, the two conic matrices assume the form


$$\begin{array}{c} C_{1}^{\prime }\propto H^{T}C_{1}H\propto [\begin{array}{ccc} A_{1}^{\prime } & 0 & 0\\ 0 & C_{1}^{\prime } & 0\\ 0 & 0 & F_{1}^{\prime } \end{array}]\; \end{array}$$ (16)

and


$$\begin{array}{c} C_{2}^{\prime }\propto H^{T}C_{2}H\propto [\begin{array}{ccc} A_{2}^{\prime } & 0 & 0\\ 0 & C_{2}^{\prime } & 0\\ 0 & 0 & F_{2}^{\prime } \end{array}] \end{array}$$ (17)

so that the conic equations are, respectively


$$\begin{array}{c} A_{1}^{\prime }x^{\prime 2}+C_{1}^{\prime }y^{\prime 2}+F_{1}^{\prime }=0\;\text{and}\;A_{2}^{\prime }x^{\prime 2}+C_{2}^{\prime }y^{\prime 2}+F_{2}^{\prime }=0 \end{array}$$ (18)

Their intersection is now easily found. From the left equation in Eq. (18) we have


$$\begin{array}{c} y^{\prime 2}=-F_{1}^{\prime }/C_{1}^{\prime }-(A_{1}^{\prime }/C_{1}^{\prime })x^{\prime 2} \end{array}$$ (19)

that, substituted into the right equation in Eq. (18), gives


$$\begin{array}{c} \left(A_{2}^{\prime }-A_{1}^{\prime }C_{2}^{\prime }/C_{1}^{\prime }\right)x^{\prime 2}=F_{1}^{\prime }C_{2}^{\prime }/C_{1}^{\prime }-F_{2}^{\prime } \end{array}$$ (20)

that is


$$\begin{array}{c} x^{\prime 2}=\frac{F_{1}^{\prime }C_{2}^{\prime }-F_{2}^{\prime }C_{1}^{\prime }}{A_{2}^{\prime }C_{1}^{\prime }-A_{1}^{\prime }C_{2}^{\prime }} \end{array}$$ (21)

Using Eq. (19) we can similarly write down an expression for $y^{\prime 2}$


$$\begin{array}{c} y^{\prime 2}=\frac{F_{2}^{\prime }A_{1}^{\prime }-F_{1}^{\prime }A_{2}^{\prime }}{A_{2}^{\prime }C_{1}^{\prime }-A_{1}^{\prime }C_{2}^{\prime }} \end{array}$$ (22)

The four points of intersection (in the transformed space) are then found as


$$\begin{array}{c} s_{a,b}^{\prime }\propto [\begin{array}{c} \pm \sqrt{x^{\prime 2}}\\ \pm \sqrt{y^{\prime 2}}\\ 1 \end{array}]\;s_{c,d}^{\prime }\propto [\begin{array}{c} \pm \sqrt{x^{\prime 2}}\\ \mp \sqrt{y^{\prime 2}}\\ 1 \end{array}] \end{array}$$ (23)

and we may convert them to the original coordinate system through


$$\begin{array}{c} s_{j}\propto Hs_{j}^{\prime }\;j=a,b,c,d \end{array}$$ (24)

The procedure leading to the solution in Eq. (24) provides the intersection points of any pair of conics in general position. The special cases of conics tangent in one or two points can be easily identified and handled accordingly.

### Two conics with two tangency points

Two conics do not always have a unique common self-polar triangle [13]. This occurs when two conics are tangent in two points. This case does not present a problem for the self-polar triangle method, as the number of common self-polar triangles is infinite and the algorithm continues to be valid. However, care must be taken in selecting the vertices of the self-polar triangle in a computational setting. Since all of the common self-polar triangles share one side, the output of the eigenvalue problem will necessarily provide two points on the common side. It is not necessarily true that the third point is the remaining vertex of the self-polar triangle. This issue can be easily solved by discarding one of the points of the shared side, and replacing it with the pole of the line joining the other two points.

### Two conics with one tangency point

The other exception is represented by the case of two conics being tangent at one point (the other two intersections being either complex conjugates or real). In this case, the conics do not have any common self-polar triangle [13], as two of its vertices coincide, making the matrix on the left-hand side of Eq. (10) singular. When this happens, however, the two coinciding vertices lie at the double intersection point. This information may be used to determine the remaining points.

Assume that during the construction of the self-polar triangle we encounter the situation where two vertices coincide. In this case, we can set $p_{1}$ as one of those vertices, and re-define the other two reference points. In particular, we can choose $p_{2}$ and $p_{3}$ on the conic, and construct $p_{0}$ as the meet of their polar lines. The effect of this mapping is to transform the equation of the conic $C_{1}$ to the parabola $y^{\prime }=x^{\prime 2}$. The two points on the conic need not to be real, so we may obtain them by intersecting any line $u\in {\mathbb{P}}^{2}$ with the conic. For example, intersecting the conic with one of the coordinate axes, we obtain


$$\begin{array}{c} p_{2,3}\propto [\begin{array}{c} 0\\ \frac{1}{2C_{1}}\left(-E_{1}\pm \sqrt{E_{1}^{2}-4C_{1}F_{1}}\right)\\ 1 \end{array}] \end{array}$$ (25)

Now, we can determine the point $p_{0}$ using


$$\begin{array}{c} p_{0}\propto \left(C_{1}p_{2}\right)\times \left(C_{1}p_{3}\right) \end{array}$$ (26)

This choice of points yields the matrix H that transforms the conic $C_{1}$ to


$$\begin{array}{c} C_{1}^{\prime }\propto H^{T}C_{1}H\propto [\begin{array}{ccc} 2 & 0 & 0\\ 0 & 0 & -1\\ 0 & -1 & 0 \end{array}] \end{array}$$ (27)

while the second matrix will transform such that $B_{2}^{\prime }=C_{2}^{\prime }=0$. The remaining two intersection points are obtained by solving the quadratic equation


$$\begin{array}{c} (A_{2}^{\prime }+E_{2}^{\prime })x^{\prime 2}+D_{2}^{\prime }x^{\prime }+F_{2}^{\prime }=0 \end{array}$$ (28)

that give


$$\begin{array}{c} x^{\prime }=\frac{-D_{2}^{\prime }\pm \sqrt{{D_{2}^{\prime }}^{2}-4F_{2}^{\prime }(A_{2}^{\prime }+E_{2}^{\prime })}}{2\left(A_{2}^{\prime }+E_{2}^{\prime }\right)} \end{array}$$ (29)

### Algorithm summary

A summary of the algorithm is provided in Algorithm 1, which reports all the steps that need to be considered in the implementation phase. Notice how the generic algorithm itself is limited to just a few lines of code, with most the steps being reserved to the handling of special conditions. A version of this algorithm is now available in the open-source SONIC toolkit [20].

**Algorithm 1 Pseudocode for conic intersection**

1: **given** conics $C_{1}$ and $C_{2}$

2: compute the eigenvectors $p_{0,1,2}$ of $C_{2}^{*}C_{1}$     ▷ Eq. (15)

3: **if** $p_{l}^{T}C_{1,2}p_{l}=p_{m}^{T}C_{1,2}p_{m}=0$ for some l≠m **then**     ▷ One tangency point

4:   set $p_{1}=p_{l}$, select $p_{2}$, $p_{3}$ on the conic     ▷ Eq. (25)

5:   compute $p_{0}\propto (C_{1}p_{1})\times (C_{1}p_{2})$     ▷ Eq. (26)

6:   set $\lambda_{3}=1$, compute $\lambda_{0,1,2}$     ▷ Eq. (10)

7:   compute H     ▷ Eq. (8)

8:   compute $C_{2}^{\prime }\propto H^{T}C_{2}H$

9:   compute $x_{a,b}^{\prime }$     ▷ Eq. (29)

10:   compute $y_{a,b}^{\prime }={x_{a,b}^{\prime }}^{2}$

11:   set ${s_{a}^{\prime }}^{T}\propto [x_{a}\;y_{a}\;1]$, ${s_{b}^{\prime }}^{T}\propto [x_{b}\;y_{b}\;1]$

12:   evaluate $s_{a,b}\propto Hs_{a,b}^{\prime }$

13:   **output** intersection points $p_{1},s_{a,b}$

14: **else if** $p_{l}\times C_{1}^{*}\left(p_{m}\times p_{n}\right)\text{≠}0\text{ for some }l\text{≠}m\text{≠}n$ **then**     ▷ Two tangency points

15: set $p_{l}=C_{1}^{*}\left(p_{m}\times p_{n}\right)$

16: **end if**

17: set $\lambda_{3}=1$ and $p_{3}$ such that ${\left(p_{l}\times p_{m}\right)}^{T}p_{3}\text{≠}0$ for l,m∈{0,1,2}

18: compute $\lambda_{0,1,2}$     ▷Eq. (10)

19: compute H     ▷ Eq. (8)

20: compute $C_{1}^{\prime }$ and $C_{2}^{\prime }$     ▷ Eq. (16)

21: compute $x^{\prime 2}$ and $y^{\prime 2}$ ▷ Eq. (21), Eq. (22)

22: compute $s_{j}^{\prime }$, *j*=*a*,*b*,*c*,*d*     ▷ Eq. (23)

23: compute $s_{j}\propto Hs_{j}^{\prime }$, *j*=*a*,*b*,*c*,*d*     ▷ Eq. (24)

24: **output** intersection points $s_{j}$, *j*=*a*,*b*,*c*,*d*

### Comparison with the classic approach

It is insightful to compare the algorithm described in this work with a common solution from the literature. Since both algorithms analytically solve the same problem and produce the same result, our comparison focuses on the algorithmic differences between the two.

Supposing we wish to intersect the conics described by $C_{1}$ and $C_{2}$, the classic solution begins by solving the cubic equation


$$\begin{array}{c} \det\left(C_{1}+\mu C_{2}\right)=0 \end{array}$$ (30)

for μ. Each of the three roots of this cubic equation describes a degenerate conic with locus


$$\begin{array}{c} C_{\mu }\propto C_{1}+\mu C_{2} \end{array}$$ (31)

which will pass through the points of intersection of the conics $C_{1}$ and $C_{2}$. Thus, since a degenerate conic consists of a pair of lines, the degenerate conic $C_{\mu }$ may be split into two lines g and h. The desired points are provided by the intersection of these two lines with one of the original conics.

If g and h are real and distinct, they can be determined utilizing the procedure outlined in Ref. [7]. This procedure begins by letting $C^{*}\propto C_{\mu }^{-1}$ be the adjugate of the degenerate conic locus $C_{\mu }$. If *i* is the index of a non-zero diagonal entry of $C^{*}$, we can define the vector $k=C_{i}^{*}/C_{ii}^{*}$, where $C_{i}^{*}$ is the *i*-th column of $C^{*}$ and $C_{ii}^{*}$ is the *i*-th diagonal element. Then, we can construct the matrix $A=C_{\mu }+[k\times ]$ and select the two lines g and h as any non-zero row and column of A.

Moreover, as discussed within Ref. [7], this technique requires direct inspection of the entries of the degenerate conic matrix and is not universally valid. Specifically, the splitting procedure is not applicable when the degenerate conic consists of a double line, or a double point (i.e., two complex conjugate lines). In this case, either (1) a different conic splitting technique needs to be employed or (2) the algorithm should select a different solution of Eq. (30) for the construction of the matrix in Eq. (31).

Other techniques for splitting a conic are not necessarily more appealing in terms of number of computations. For example, we could define a change of coordinates for transforming the degenerate conic to a standard expression consisting of well-known degenerate lines, and determine the lines in the original coordinates by reverting the transformation (e.g., see Ref. [20]). However, since it is impossible to define a splitting operation without exceptions [7], all such procedures require handling of multiple conditional statements that considerably lengthen software implementations.

We emphasize that returning to Eq. (30) and selecting a different root for of μ is straightforward and it is possible that choosing another root will remove the necessity of handling multiple special cases. However, the identification of these special cases is not as straightforward as in the case of the solution based on the common self-polar triangle. Indeed, the self-polar triangle approach identifies the special cases directly in terms of the relative position of the two conics. This is done through direct residuals evaluation. Differently, the classic solution identifies special cases in terms of the relative position of auxiliary products (the degenerate lines) and allows the identification of such special cases only after many steps into the process of splitting the conic.

Numerical experiments demonstrate that the runtimes of the classical method and the proposed method are similar, with the self-polar triangle method usually being slightly faster. Moreover, the self-polar triangle method provides more geometric clarity, limits the calculation of parameters to only those necessary for solving the problem, and has fewer special cases requiring special treatment.

## Numerical example

Three short experiments will show the application of the proposed technique for conic intersection to three different image analysis scenarios. A numerical experiment of the behavior of the algorithm near the tangency conditions is also provided. Algorithm runtime results are provided for each of these experiments.

### Conic intersection for crater identification

**Fig 2:**
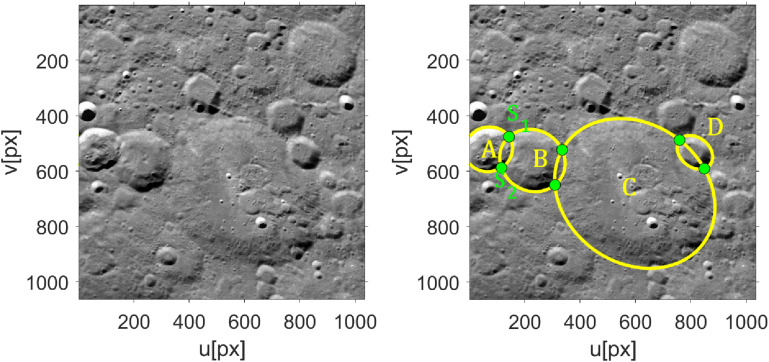
Craters on the lunar surface, with their elliptical approximation and the real intersection points. Product ID LUB0280B.243 [21].

Fig 2 shows a picture of a portion of lunar surface captured during the Clementine mission [22]. In the context of image-based spacecraft terrain relative navigation (TRN), the points of intersection of two or more craters (approximated as ellipses) in the image can be used to construct their invariants [4]. The calculation of the invariants is a key step in matching craters in the image to craters in a catalog. Once the desired craters have been fitted with conics, we can perform conic intersection using the self-polar triangle method.

As an example, consider the conic matrices of the craters labeled with *A* and *B* in Fig 2:


$$\begin{array}{c} C_{A}\propto [\begin{array}{ccc} 0.0027614 & -0.00025812 & -0.48308\\ -0.00025812 & 0.0030422 & -1.6516\\ -0.48308 & -1.6516 & 1000 \end{array}] \end{array}$$

$$\begin{array}{c} C_{B}\propto [\begin{array}{ccc} 0.0032096 & 0.00013804 & -0.29495\\ 0.00013804 & 0.0036668 & -1.923\\ -0.29495 & -1.923 & 1000 \end{array}] \end{array}$$

Applying the steps summarized in Algorithm 1, we can calculate the homography matrix


$$\begin{array}{c} H=[\begin{array}{ccc} -0.0646+1.0015i & -0.0646-1.0015i & 0.1303\\ 0.2021+1.7804i & 0.2021-1.7804i & -0.4031\\ 0.0004+0.0030i & 0.0004-0.0030i & 0.0001 \end{array}] \end{array}$$

which transforms the conic matrices to


$$\begin{array}{c} C_{1}^{\prime }\propto [\begin{array}{ccc} 0.29+0.48i & 0 & 0\\ 0 & 0.29-0.48i & 0\\ 0 & 0 & 0.43 \end{array}] \end{array}$$

$$\begin{array}{c} C_{2}^{\prime }\propto [\begin{array}{ccc} 0.14-0.40i & 0 & 0\\ 0 & 0.14+0.40i & 0\\ 0 & 0 & 0.71 \end{array}] \end{array}$$

Proceeding with the algorithm as described, we obtain the following real and complex intersection points


$$\begin{array}{c} s_{1}\propto [\begin{array}{c} 142.84\\ 476.40\\ 1 \end{array}]\;s_{2}\propto [\begin{array}{c} 115.25\\ 588.6\\ 1 \end{array}] \end{array}$$

$$\begin{array}{c} s_{3}\propto [\begin{array}{c} 10.18-1224.70i\\ -623.67+101.38i\\ 1 \end{array}]\;s_{4}\propto [\begin{array}{c} 10.18+1224.70i\\ -623.67-101.38i\\ 1 \end{array}] \end{array}$$

The reader may verify with a simple calculation that these points belong to the conics.

It is useful to compare the results of our method with the more classical conic intersection approach outlined in Ref. [7]. First, as expected, the numerical solution produced by both procedures is the same to within machine precision. Second, we observe the runtimes to be similar, but with the newly proposed method being slightly faster. Over 100,000 repetitions of this experiment, the average runtime obtained with the self-polar triangle method presented in this work was 0.027 ms, while the average runtime of the classic technique from Ref. [7] was 0.033 ms. The latter runtime depends on the specific implementation chosen for the conic splitting technique. The value reported was obtained implementing the splitting algorithm also utilized in the SONIC software [20], which guarantees that two lines are determined independently of their relative position.

### Conic intersection for iris detection

When performing iris detection for face recognition, only the portion of the iris which is not covered by the eyelid should be considered [23], and pictures with small percentages of visible iris should be discarded [24]. In order to identify usable portions, however, the intersection between the iris and the eyelid needs to be determined first.

**Fig 3:**
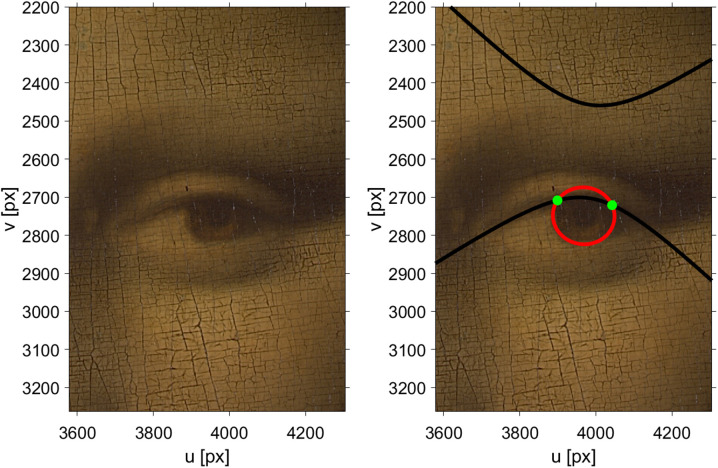
Left: zoom on the eye of the Monna Lisa painting from Leonardo Da Vinci, retrieved from *Wikimedia Commons* [26]. Right: overlay of the fitted conics (iris in red, eyelid in black). The pair of real conic intersection points are shown in green.


While the iris is usually considered circular, it may appear as elliptical in non-frontal pictures. Similarly, the eyelid is often approximated with a quadratic equation [25]. Therefore, determining the portion of visible iris requires nothing more than determining the intersection of two conics. For example, consider the right eye of Lisa Gherardini, in the Mona Lisa painting from Leonardo Da Vinci [26] in Fig 3. Points were sampled on the contour of the iris and upper eyelid and translated by the overall mean value of their coordinates to guarantee better numerical behavior. The translation was, respectively, of 3951.4 px and 2755.6 px for the *u* and *v*-coordinates. Conics were fit to these points using a simple least-squares approach. The resulting conics are shown on the right in Fig 3, and the corresponding conic matrices are

$$\begin{array}{c} C_{iris}\propto [\begin{array}{ccc} -0.050692 & 0.0010064 & 0.8503\\ 0.0010064 & -0.059349 & -0.41593\\ 0.8503 & -0.41593 & 315.55 \end{array}] \end{array}$$

$$\begin{array}{c} C_{eyelid}\propto [\begin{array}{ccc} -0.030099 & -0.0069514 & -0.24664\\ -0.0069514 & 0.046108 & 8.3242\\ -0.24664 & 8.3242 & 773.85 \end{array}] \end{array}$$

which provide the following real and complex intersection points


$$\begin{array}{c} s_{1}\propto [\begin{array}{c} -52.30\\ 47.19\\ 1 \end{array}]\;s_{2}\propto [\begin{array}{c} 91.19\\ -34.73\\ 1 \end{array}] \end{array}$$

$$\begin{array}{c} s_{3}\propto [\begin{array}{c} 31.37-149.04i\\ -163.09-14.49i\\ 1 \end{array}]\;s_{4}\propto [\begin{array}{c} 31.37+149.04i\\ -163.09+14.49i\\ 1 \end{array}] \end{array}$$

The two real-valued intersection points $s_{1}$ and $s_{2}$ correspond to the green dots in Fig 3 after translation.

As before, the proposed method and the classical method from Ref. [7] produce the same solution to within numerical precision. The runtime trends are also the same. Over 100,000 repetitions of this experiment, the average runtime obtained with the self-polar triangle method presented in this work was 0.029 ms, while the average runtime of the classic technique from Ref. [7] was 0.038 ms.

Notice that at this point the center c of the iris can be easily identified as pole of the line at infinity [12]


$$\begin{array}{c} c\propto C_{iris}^{*}[\begin{array}{c} 0\\ 0\\ 1 \end{array}]\propto [\begin{array}{c} 16.64\\ -6.73\\ 1 \end{array}] \end{array}$$ (32)

enabling easy identification of the angles between the intersection points and the horizontal axis, utilized in iris identification techniques.

### Conic tangency in images of bubbles

It is not uncommon to observe bubbles in images of water and other fluids. These bubbles are often ellipsoidal in shape and their contours project to ellipses in an image. In this example we illustrate the utility of the proposed conic intersection algorithm for computing the tangency points between pairs two bubbles. Our specific example uses images of bubbles produced during the electrolysis of water in microgravity [27].

**Fig 4:**
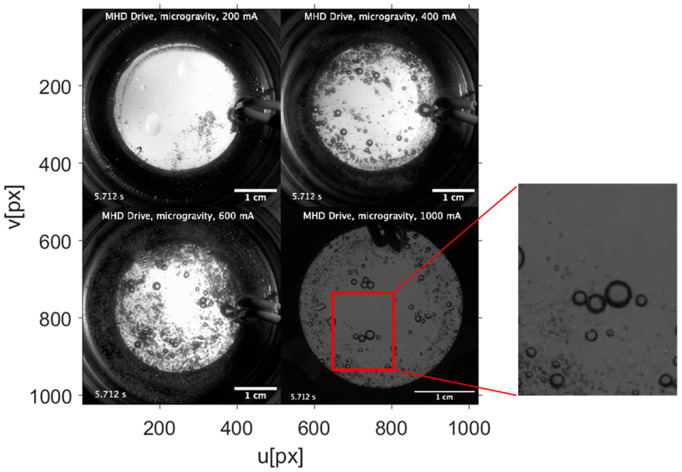
Example frame from Supplementary Video 8 of Ref. [27] showing oxygen and hydrogen bubbles generated in a magenetohydrodynamic water electrolysis cell for microgravity operation. In the lower right view, three bubbles are tangent to each other.

**Fig 5:**
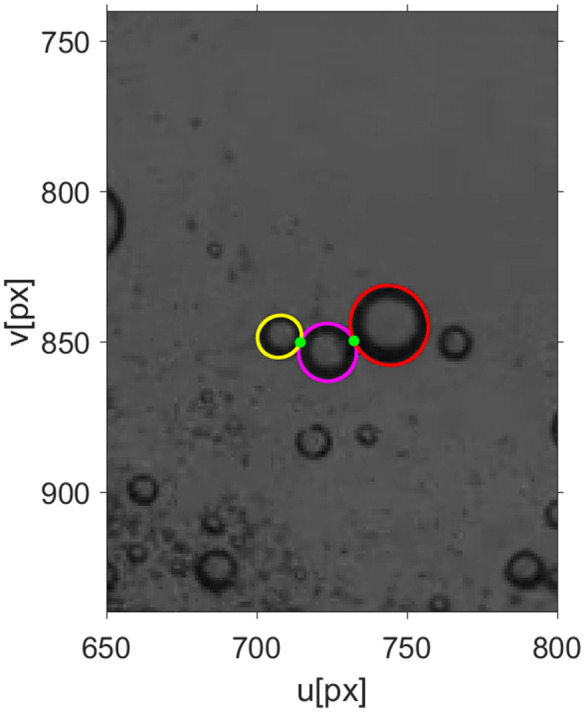
Points of tangency of three bubbles from Fig 4.


Consider, for example, the image from Supplementary Video 8 of Ref. [27] shown in Fig 4. After applying a translation of (728.44, 848.19) pixels to recenter the bubbles and improve numerical conditioning, the conic matrices (from left to right in Fig 5) are:

$$\begin{array}{c} C_{A}\propto [\begin{array}{ccc} -0.2584 & -0.0160 & -5.3854\\ -0.0160 & -0.2809 & -0.3158\\ -5.3854 & -0.3158 & -98.4089 \end{array}] \end{array}$$

$$\begin{array}{c} C_{B}\propto [\begin{array}{ccc} -0.2200 & 0.0020 & -1.0801\\ 0.0020 & -0.2222 & 1.1951\\ -1.0801 & 1.1951 & 8.8317 \end{array}] \end{array}$$

$$\begin{array}{c} C_{C}\propto [\begin{array}{ccc} -0.1840 & 0.0067 & 2.9106\\ 0.0067 & -0.1763 & -0.7492\\ 2.9106 & -0.7492 & -17.5135 \end{array}] \end{array}$$

The primes on the conic matrices indicate the use of shifted coordinates. The points of intersection for the left and middle conics ***C***_*A*_ and $C_{B}$ are


$$\begin{array}{c} s_{1,2}=[\begin{array}{c} 24.85-214.46i\\ -208.01-31.49i\\ 1 \end{array}]\;s_{3,4}\propto [\begin{array}{c} -13.94\\ 2.01\\ 1 \end{array}] \end{array}$$

while the points of intersection between the middle and the right conic $C_{B}$ and $C_{C}$ are


$$\begin{array}{c} s_{1,2}\propto [\begin{array}{c} 542.78\pm 236.59\\ -225.13\pm 547.01\\ 1 \end{array}]\;s_{3,4}\propto [\begin{array}{c} 3.94\\ 1.49\\ 1 \end{array}] \end{array}$$

Adding the (728.44, 848.19) pixels back to these intersection points yields tangency points of


$$\begin{array}{c} s_{AB}\propto [\begin{array}{c} 714.50\\ 850.20\\ 1 \end{array}]\;s_{BC}\propto [\begin{array}{c} 732.38\\ 849.68\\ 1 \end{array}] \end{array}$$

Over 100,000 repetitions of this experiment, the average runtime of the self-polar triangle method presented in this work was 0.076 ms, while the average runtime of the classic technique from Ref. [7] approach was 0.1 ms.

### Algorithm behavior near tangency conditions

It is interesting to study the behavior of the proposed algorithm near the special case of a pair of conics with one or two tangency points. In particular, we may track the intersection points on the complex plane as the pair of conics transition from no physical intersection (i.e., complex-valued points) to tangency (i.e., repeated real-valued points) to distinct physical intersections (i.e., distinct real-valued points). This is done through two experiments. In each experiment we construct a root locus that tracks how the common roots of the polynomial equations evolve as the conics’ relative position changes.

**Fig 6:**
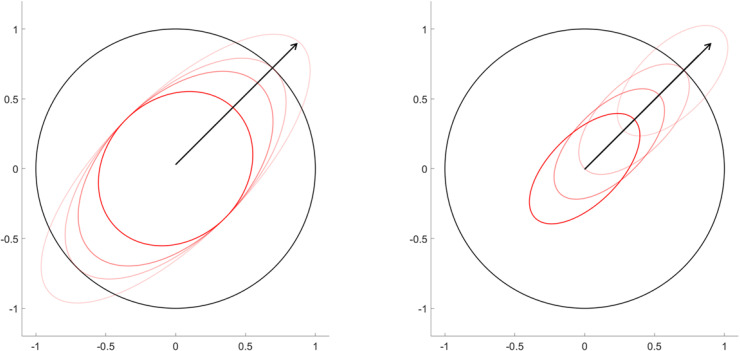
Configuration of two conics used in the study of the algorithm behavior near the edge cases of two (left image) and one (right image) tangency points.

**Fig 7:**
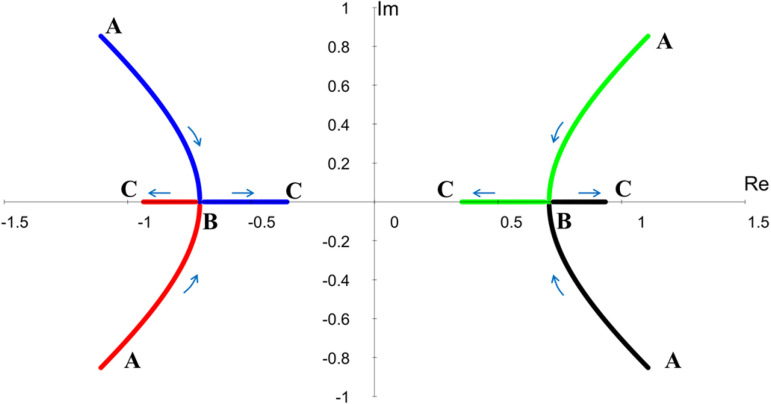
Root locus of the x-component of the four intersection points near the edge case of two tangency points. **A,** four complex intersections; **B,** two tangency points; **C,** four real (distinct) intersections.

In the first experiment, we consider a circle and an ellipse that share a common center. The experiment begins with the ellipse contained entirely within the circle. From this starting point, the major axis of the ellipse is gradually increased, as shown on the left in Fig 6. During the process, we draw the location of the first component of the four intersection points in the complex plane in Fig 7. At the beginning of the experiment, there is no intersection and we have a pair of complex conjugate roots (condition A). Then, as the ellipse grows and the two conics become tangent at two points, we the complex conjugate roots merge to form a repeated root on the real axis (condition B). Finally, as the major axis of the ellipse is increased further (with the two conics having four real distinct intersections) the two roots separate into two distinct points on the real axis (condition C). This shows a smooth transition from the generic case of four distinct intersection points to that of two coinciding ones, and vice-versa. The average runtime for this experiment was of 0.012 ms. Using the classic approach, the average runtime was of 0.041 ms.

**Fig 8:**
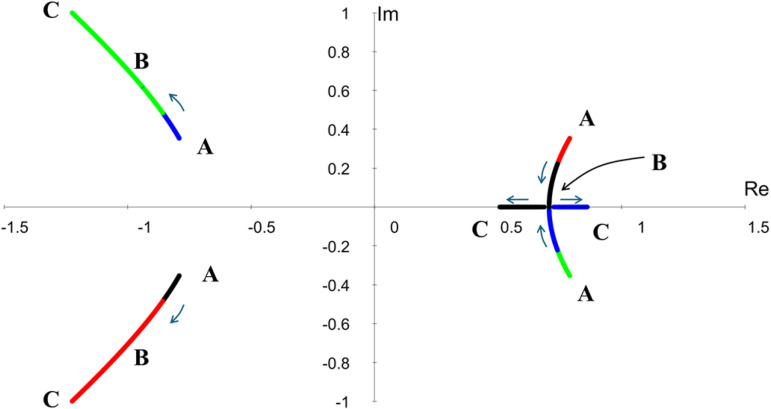
Root locus of the x-component of the four intersection points near the edge case of one tangency point. **A,** four complex intersections; **B,** one tangency points and two complex intersections; **C,** two real (distinct) intersections and two complex intersections.

In the second experiment, we translate the internal ellipse in a direction parallel to its major axis, as shown on the right in Fig 6. The complex location of its intersection points is shown in Fig 8. As before, we begin with no physical intersection, corresponding to a pair of complex conjugate roots (condition A). When the two conics become tangent at one point, the two complex conjugate roots again merge into a repeated root on the real axis (condition B). Finally, as the conic is translated further, the repeated roots split into two distinct real roots (condition C). Once again, we observe a smooth transition from four distinct intersection points to a double point, and vice-versa. This suggests that the algorithm effectively handles transitions between edge cases without introducing unexpected behavior of the roots. The average runtime was of 0.026 ms compared with the 0.052 ms of the classic approach.

## Conclusions

Finding the intersection of two conics is a classical problem arising in science, engineering, and art. It is particularly pervasive in situations where image analysis is required. Although techniques already exist to solve this problem, a straightforwardly-implementable algorithm may also be built by applying a change of projective coordinates using the properties of self-polar triangles. The resulting algorithm requires the solution of a degree-three eigenvalue problem (i.e., a cubic equation), followed by a simple quadratic equation. As compared to more well-known methods, the self-polar triangle method results in simpler code. The self-polar triangle method is also easy to implement in environments without access to extensive software libraries.

## Supporting information

S1 File Experimental results setup. (PDF)

## References

[1] Kepler J. Astronomia nova, seu physica coelestis, tradita commentariis de motibus stellae Martis ex observationibus G.V. Tychonis Brahe. Heidelberg: E. Vögelin. 1609.

[2] Curtis HD. Orbital Mechanics for Engineering Students. 4th Ed. Cambridge, MA: Butterworth-Heinemann. 2020.

[3] Robbins SJ. A New Global Database of Lunar Impact Craters >1–2 km: 1. Crater Locations and Sizes, Comparisons With Published Databases, and Global Analysis. Journal of Geophysical Research: Planets. 2019;124(4):871–92.

[4] Christian JA, Derksen H, Watkins R. Lunar Crater Identification in Digital Images. The Journal of the Astronautical Sciences. 2021;68:1056–144. 35001965 10.1007/s40295-021-00287-8 PMC8692363

[5] Kaminski JY, Knaan D, Shavit A. Single image face orientation and gaze detection. Machine Vision and Applications. 2009;21(85).

[6] Ding Y, Yang J, Larsson K, Olsson C, Åström K. Revisiting the P3P problem. In: Proceedings of the IEEE/CVF Conference on Computer Vision and Pattern Recognition, 2023.

[7] Richter-Gebert J. Perspectives on projective geometry. A guided tour through real and complex geometry. Heidelberg: Springer. 2011.

[8] Faucette WM. A geometric interpretation of the solution of the general quartic polynomial. The American Mathematical Monthly. 1996;103:51–7.

[9] Bose NK. In: Gröbner Bases: An Algorithmic Method in Polynomial Ideal Theory. Gröbner Bases: An Algorithmic Method in Polynomial Ideal Theory. Dordrecht: Springer Netherlands. 1995. 89–127.

[10] Chomicki C, Breuils S, Biri V, Nozick V. Intersection of Conic Sections Using Geometric Algebra. Lecture Notes in Computer Science. 2004.

[11] Cardano G. Ars Magna, Sive de Regulis Algebraicis. Nuremberg: Johannes Petreius. 1545.

[12] Semple JG, Kneebone GT. Algebraic Projective Geometry. Oxford, UK: Oxford University Press. 1952.

[13] Woods FS. An introduction to advanced methods in analytic geometry. Boston: Ginn and Company. 1992.

[14] Huang H, Zhang H, Cheung YM. The common self-polar triangle of separate circles: properties and applications to camera calibration. In: Proc. IEEE Int. Conf. Image Process., Phoenix, AZ, USA, 2016. 1170–4. 10.1109/ICIP.2016.7532542

[15] Huang H, Zhang H, Cheung Y-M. The common self-polar triangle of concentric circles and its application to camera calibration. In: Proc. IEEE Conf. Comput. Vis. Pattern Recognit. (CVPR), Boston, MA, USA, 2015. 4065–72. 10.1109/CVPR.2015.7299033

[16] Zhang Q, Wang Q. Common Self-polar Triangle of Concentric Conics for Light Field Camera Calibration. Lecture Notes in Computer Science. Springer International Publishing. 2019. 18–33. 10.1007/978-3-030-20876-9_2

[17] Huang H, Zhang H, Cheung Y-M. Homography Estimation from the Common Self-Polar Triangle of Separate Ellipses. In: Proceedings of the IEEE Conference on Computer Vision and Pattern Recognition (CVPR), Las Vegas, NV, USA, 2016. 1737–44. 10.1109/CVPR.2016.192

[18] Aronhold S. Über eine fundamentale Begründung der Invariantentheorie. Journal für die Reine und Angewandte Mathematik. 1863.

[19] Hesse O. Vorlesungen aus der analytischen Geometrie der Kegelschnitte. Zeitschrift für Mathematik und Physik. 1876.

[20] Thrasher AC, Krause M, Henry S, Mancini M, Sonin P, Christian JA. SONIC: Software for Optical Navigation and Instrument Calibration. The Journal of Open Source Software. 2024;9(101):6916. doi: 10.21105/joss.06916

[21] PDS data products. Clementine EDR image archive. https://planetarydata.jpl.nasa.gov/img/data/clementine/cl_0055/lun243/luxxxxxx/luxxxxxb/

[22] Nozette S, Rustan P, Pleasance LP, Kordas JF, Lewis IT, Park HS, et al. The clementine mission to the moon: scientific overview. Science. 1994;266(5192):1835–9. doi: 10.1126/science.266.5192.1835 17737076

[23] Perez CA, Lazcano VA, Estevez PA. Real-Time Iris Detection on Coronal-Axis-Rotated Faces. IEEE Trans Syst, Man, Cybern C. 2007;37(5):971–8. doi: 10.1109/tsmcc.2007.900647

[24] Daugman J. How Iris Recognition Works. IEEE Trans Circuits Syst Video Technol. 2004;14(1):21–30. doi: 10.1109/tcsvt.2003.818350

[25] Maseedupally V, Gifford P, Swarbrick H. Variation in normal corneal shape and the influence of eyelid morphometry. Optom Vis Sci. 2015;92(3):286–300. doi: 10.1097/OPX.0000000000000511 25654494

[26] Da Vinci L. Mona Lisa. 1503–1506. https://commons.wikimedia.org/wiki/File:Mona_Lisa,_by_Leonardo_da_Vinci,_from_C2RMF.jpg

[27] Akay Ö, Monfort-Castillo M, St Francis T, Becker J, Saravanabavan S, Romero-Calvo Á, et al. Magnetically induced convection enhances water electrolysis in microgravity. Nat Chem. 2025;17(11):1673–9. doi: 10.1038/s41557-025-01890-0 40826233 PMC12580345

