# Supplementary material for: Easy conic intersection with the common self-polar triangle
Source: PLoS One. 2026 Jul 17;21(7):e0340348. doi: 10.1371/journal.pone.0340348 (PMC13379144; doi:10.1371/journal.pone.0340348)
Supplement: S1 File — (PDF) [file pone.0340348.s001.pdf]

S1 Supporting Information

1 Algorithm behavior near the tangency condition: conics parameters

The configuration plotted on the left-hand side of Fig. 4 in the manuscript was obtained utilizing a circle and an ellipse of varying semi-major axis length. The circle has unit radius and is centered at the origin. The ellipse is centered at the origin, and has tilt angle ψ and semi-minor axis b given by

$$\psi = 45 \text{ deg} \qquad b = 0.5$$

The semi-major axis a is sampled from the interval $[0.6, 1.6]$ such that 1201 equally spaced values are obtained.

The configuration plotted on the right-hand side of Fig. 4 is obtained utilizing a unit circle centered at the origin, and an ellipse characterized by

$$\psi = 45 \text{ deg} \qquad a = 0.5 \qquad b = 0.25$$

The x-component of the center is sampled in the interval $[0, 1]$. The center is constrained to lie on the line $x - y = 0$.
